# Improved cell composition deconvolution method of bulk gene expression profiles to quantify subsets of immune cells

**DOI:** 10.1186/s12920-019-0613-5

**Published:** 2019-12-20

**Authors:** Yen-Jung Chiu, Yi-Hsuan Hsieh, Yen-Hua Huang

**Affiliations:** 1 Institute of Biomedical Informatics, National Yang-Ming University, No.155, Sec. 2, Li-Nong St., Beitou Dist, Taipei, 11221 Taiwan; 2 Centre for Systems and Synthetic Biology, National Yang-Ming University, Taipei, 11221 Taiwan

**Keywords:** Deconvolution, Immune cells, Bulk gene expression profiles

## Abstract

**Background:**

To facilitate the investigation of the pathogenic roles played by various immune cells in complex tissues such as tumors, a few computational methods for deconvoluting bulk gene expression profiles to predict cell composition have been created. However, available methods were usually developed along with a set of reference gene expression profiles consisting of imbalanced replicates across different cell types. Therefore, the objective of this study was to create a new deconvolution method equipped with a new set of reference gene expression profiles that incorporate more microarray replicates of the immune cells that have been frequently implicated in the poor prognosis of cancers, such as T helper cells, regulatory T cells and macrophage M1/M2 cells.

**Methods:**

Our deconvolution method was developed by choosing ε-support vector regression (ε-SVR) as the core algorithm assigned with a loss function subject to the *L1*-norm penalty. To construct the reference gene expression signature matrix for regression, a subset of differentially expressed genes were chosen from 148 microarray-based gene expression profiles for 9 types of immune cells by using ANOVA and minimizing condition number. Agreement analyses including mean absolute percentage errors and Bland-Altman plots were carried out to compare the performances of our method and CIBERSORT.

**Results:**

In silico cell mixtures, simulated bulk tissues, and real human samples with known immune-cell fractions were used as the test datasets for benchmarking. Our method outperformed CIBERSORT in the benchmarks using in silico breast tissue-immune cell mixtures in the proportions of 30:70 and 50:50, and in the benchmark using 164 human PBMC samples. Our results suggest that the performance of our method was at least comparable to that of a state-of-the-art tool, CIBERSORT.

**Conclusions:**

We developed a new cell composition deconvolution method and the implementation was entirely based on the publicly available R and Python packages. In addition, we compiled a new set of reference gene expression profiles, which might allow for a more robust prediction of the immune cell fractions from the expression profiles of cell mixtures. The source code of our method could be downloaded from https://github.com/holiday01/deconvolution-to-estimate-immune-cell-subsets.

## Background

Tumors are heterogeneous systems containing not only cancer cells but also a variety of other types of genetically normal cells. Those non-cancer cells are not just unimportant players, and accumulating evidence has revealed that certain types of immune cells infiltrating in the tumor microenvironment (TME) may actively interact with cancer cells and thus promote their malignant phenotypes such as enhancing survival of cancer cells and supporting their metastasis (for review see [1, 2]).

Modulating the tumor immunity could be an effective strategy to treat cancers. The success of checkpoint blockade therapy against CTLA4 or PD1/PDL1 suggests that immunotherapy of cancers is promising and the effect could be fairly durable, at least for some patients with certain tumors. Therefore, more in-depth studies might help resolve questions such as finding the distinctive expression features of each type of immune cells, and the association between patients’ immunophenotypes and responses to differential anticancer treatments.

To further study cancer immunity, quantifying the composition of the immune infiltrates in cancer tissues is an important issue. Apart from pure experimental approaches in cell biology, such as immunohistochemistry (IHC) and flow cytometry, computational approaches that can take bulk transcriptome profiling data as the input to estimate the relative abundance of each immune cell subset have been developed (for review see [3]). The underlying rationale of such computational approaches is treating bulk expression profiles as a linear combination of the expression profiles of a variety of cell subsets, and thus deconvolution of the mixture of profiles may recover the fractions of different types of cells.

A number of different methods have been proposed to perform the deconvolution of the gene expression profiles of cell mixtures, including linear least squares regressions [4, 5], non-negative matrix factorization [6–8], quadratic programming [9, 10], *v*-support vector regression [11], etc. Among the approaches, Newman et al. developed a novel tool, CIBERSORT, which can perform linear support vector regression (SVR) to deconvolute a mixture of gene expression profiles [11]. One of the benchmark experiments of CIBERSORT, which was performed by taking the samples of peripheral blood mononuclear cells (PBMC) with the fractions of cell subsets confirmed by flow cytometry, revealed a high Pearson correlation of approximately 0.5 to 0.76 between the predicted abundance and the ground truth. Furthermore, to enumerate different types of immune cells, CIBERSORT utilizes a reference set consisting of 113 gene expression profiles, which correspond to 22 types of immune cells, to construct a reference gene signature matrix LM22. This means that CIBERSORT has been designed to predict the relative proportions of diverse immune cell types.

However, there is some bias in the choice of reference gene expression profiles to build LM22, which makes CIBERSORT a tool that might not be very optimized for the purpose of deconvoluting certain immune cells that have been reported to modulate cancer immunity, such as regulatory T cells (Treg), T helper cells (Th), and macrophage M1 and M2 cells.

For example, even though in LM22 there are 7 and 8 gene expression signatures of naïve B cells and memory B cells respectively, there are only 3 signatures collected each for Treg cells and Th cells. In addition, in LM22 there are 24 microarray samples collected for monocytes and macrophage M0 cells, whereas there are only 3 microarray samples derived from macrophage M2 cells. The obviously lower number of replicates in the reference dataset for certain types of immune cells may introduce a risk of decreasing the robustness in finding the differentially expressed genes to build specific expression signatures for those cells, influencing the precision of the predicted fractions. After all, while monocytes are found in bone marrow, blood, and spleen, they differentiate into macrophages under the control of a series of cytokines when recruited into tissues (for review see [12]). In particular, tumor-associated macrophages which have been correlated with the poor prognosis of a number of cancers have been reported to express M2-like phenotypes (for review see [13–15]). Therefore, in this study, we developed an immune cell deconvolution method that might achieve a better accuracy on the expression profiles derived from complex tissue samples such as tumor masses.

We built a new dataset of reference gene expression profiles such that a higher number of microarray samples could be recruited for the immune cell types that have been reported to influence the survival of cancer patients, including Treg, Th, M1, and M2 cells. The expression levels of well-known marker genes for each of those immune cells were assessed in the microarray data. Benchmarking of our method was performed by assessing the cell composition in the microarray datasets of single-type cells, cell mixtures such as PBMC, and in silico cell mixtures.

## Methods

### Study design

**Fig. 1:**
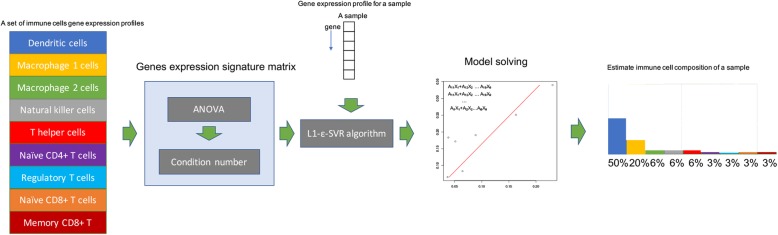
The workflow of our deconvolution method

The workflow overview of the development of our method is illustrated in Fig. 1. To construct the reference gene signature matrix to be used in regression, we surveyed the NCBI Gene Expression Omnibus (GEO) microarray datasets and thus we collected the gene expression profiles for 9 types of immune cells, including dendritic cells (DCs), macrophage M1 cells, macrophage M2 cells, natural killer cells (NK cells), naïve CD4 T cells, T helper cells, regulatory T cells, naïve CD8+ T cells, and memory CD8+ T cells. Then by using ANOVA and calculating condition numbers we chose a subset of differentially expressed genes to create the reference gene expression signature matrix. For implementation of the computational codes for our method, we took advantage of the linearSVR class in the Python package scikit-learn. We chose ε-support vector regression (ε-SVR) as the core algorithm to perform deconvolution in this study, and the *L1*-loss was applied. Our ε-SVR based deconvolution method could take bulk expression profiles with unknown cell composition to predict the fractions of 9 types of immune cells in complex tissues.

### Datasets for building the reference expression signature

We downloaded 148 microarray samples from NCBI GEO, where at least 9 replicate samples were included for each cell type. The numbers of samples for different types of immune cells are listed in Table 1 and the NCBI GEO sample accession numbers for those microarray samples are provided in Additional file 1: Table S1.

**Table 1:** The numbers of microarray samples for building the reference expression signature for 9 types of immune cells

Immune cell types	Subtypes	Treatments	Replicates
Dendritic cell	Immature		9
	Mature		7
	Tolerogenic		8
Naïve CD4 T cell	CD4+ CD45RA+		9
Naïve CD8 T cell	CD3+ CD8+		13
Memory CD8+ T cell	CD45RO+		12
Natural killer cell	IL-2 treatment	IL-2	12
Macrophage M1	IFNγ treatment	IFNγ	9
	IFNγ + TNF treatment	IFNγ + TNF	5
Macrophage M2	IL-4 treatment	IL-4	25
T helper cell	CD4 + IL-12 treatment	CD4 + IL-12	11
	CD4 + IL-4 treatment	CD4 + IL-4	12
Regulatory T cell	IFNγ treatment	IFNγ	8
	No treatment		8

### Building the gene expression signature matrix

The microarray platform of all the samples obtained for building the reference gene signatures is Affymetrix U133 plus 2 (see Additional file 2: Table S1). The microarray data were quantile normalized using the Robust Multi-array Average (RMA) procedure offered in the R package affy [16]. Analysis of variance (ANOVA) was performed using the R package stats to find genes that have significantly higher values in certain types of immune cells than in the others. The expression values of those differentially expressed genes (DEGs) were taken to build the initial matrix for the gene expression signatures of the immune cells.

To make the reference gene signature matrix more robust against input variations or noises, we used condition number as a means to determine how many DEGs should be included in the final matrix. Technically, the condition number of a matrix is the product of the norm of the matrix and the norm of its inverse. Condition number is a measurement of how sensitive a mathematical function might be to changes or errors in the input data. In numerical analysis, a function with a low value of condition number is considered to be well-conditioned, since it might have a relatively stable solution when there are small fluctuations in the input data. A number of cell deconvolution methods have applied the minimization of condition numbers to the optimization of the reference gene signature matrix [4, 9, 11].

To select the genes to generate the final gene expression signature matrix that has the minimal condition number, all of the probe sets in the raw expression signature matrix were firstly sorted by the ANOVA *p*-value in ascending order. The condition numbers for the gene expression matrix consisting of the top *G* probe sets × 148 arrays were calculated by iterating through different *G* values with a step size of 500. The R function kappa was used to estimate the condition number of each matrix.

The list of probe sets that could give the minimal condition number among all of the top lists (i.e. top 500, 1000, 1500, *...*) was taken to build the final reference gene signature matrix. After selecting the final *G* probe sets, the median expression level of each probe set for all of the replicates of one type of immune cells was estimated and thus the final gene expression signature matrix consists of column vectors for immune cell types, each column vector containing *G* values for each immune cell type. Then the R package hgu133plus2.db was used to map probe sets to human genes. Whenever multiple probes/probe sets could be mapped to one gene, they would be collapsed to just one record by using a homemade R function, where the expression value of this gene would be assigned with the probe/probe set that has the highest median value across samples.

### Assessment of the reference gene expression signature in clustering cells

To further evaluate if the final reference expression signature of genes might allow for a robust composition deconvolution of immune cells, linear discriminant analysis (LDA), which can consider class-label information, was applied to visualize the clustering of different types of immune cells in the context of the selected *G* genes by using the LinearDiscriminantAnalysis class in the Python package scikit-learn. In addition, hierarchical clustering, K-means, and weighted gene co-expression network analysis (WGCNA) [17] were carried out to determine if the cell clustering based on the expression values of the selected *G* genes agreed with the cell phenotypes.

### ε-Support vector regression

Deconvolution can be conceived as finding the solution to the convolution equation:

$$ {\mathrm{b}}_i={\mathrm{a}}_{i,1}{\mathrm{x}}_1+{\mathrm{a}}_{i,2}{\mathrm{x}}_2\dots {\mathrm{a}}_{i,j}{\mathrm{x}}_j $$

where b_*i*_ is the expression level of gene *i* in a sample of cell mixture, a_*i,j*_ is the expression level of gene *i* in cell type *j* (derived from the reference expression signature for this cell type), and x _*j*_ is the unknown proportion of cell type *j* in the cell mixture. Among the available cell composition deconvolution tools, CIBERSORT uses a ν-SVR based approach and it outperformed the other tools in benchmarking experiments [11]. Since SVR might be superior to the other regression methods in providing a sparse solution, we chose one type of SVR, ε-insensitive support vector regression (ε-SVR), as the core algorithm to perform deconvolution in this study. To implement our method, we took advantage of the linearSVR class in the Python package scikit-learn. Unlike ν-SVR that is used by CIBERSORT, ε-SVR does not control the proportion of the support vectors to use in the final model [18]. This means that an ε-SVR based deconvolution model might allow for a higher flexibility in combining different predictor variables, without setting a lower bound on support vectors. After all, for one bulk tissue without prior knowledge about its cell composition, it would be arbitrary to decide a lower bound of immune cell types. In addition, *L1*-loss function was applied in order to minimize the mean average error (MAE) between the predicted and ground truth values [19]. *L1*-loss function might make the regression model more robust to outlier values in the input data than *L2*-loss function, since *L2*-loss function leads to the much larger error for outliers because of the consideration of the squared differences.

### Benchmark of our deconvolution method

To assess the performance of our cell-type deconvolution method, a series of benchmarking approaches were used by recruiting a variety of test samples such as pure cells, in silico cell mixtures, simulated bulk tissues, and PBMC samples with flow cytometry results.

### Analysis of pure cell types

We started with the analysis of pure cell types by using a leave-one-out strategy. In each test run, one of the 148 microarray samples obtained from NCBI GEO for building the reference gene expression signature for the 9 types of immune cells was used as the test dataset to perform deconvolution, while the remaining 147 samples were put through to build the reference gene expression signature for testing by using the aforementioned ANOVA-based procedure. Box plots and bar charts were produced to show the distributions of the predicted cell-type fractions in those pure-cell samples. The results were compared with the predictions made by CIBERSORT.

### Analysis of in silico cell mixtures

To further evaluate the performance of our method in predicting the immune cell composition, we prepared expression profiles of in silico cell mixtures. In silico mixture samples of 9 types of immune cells were prepared for this benchmark. In each test run, a set of 9 microarrays, each from one unique immune cell type, were randomly sampled from the 148 microarrays presented in Table 1. The expression value for each gene in an in silico mixture sample was a randomly-weighted sum across 9 types of immune cells. The remaining 139 microarrays were used to build the gene expression signature matrix dedicated to a single testing run. Bland-Altman plots were generated by using a homemade R function in order to assess the agreement of the predicted cell fractions with the real cell-specific weights in the in silico cell mixture samples [20]. The agreement for individual tests was summarized by using the limits of agreement (LoA) technique [21]. LoA corresponds to the 95% confidence interval (CIs) computed as the mean difference ± 1.96 × standard deviation (SD) of the differences. A smaller LoA might suggest that the predicted cell fractions agree better with the real composition.

### Analysis of simulated bulk tissues

To assess if our method might be readily applied to estimate the cell composition using the bulk expression profiles derived from tissues consisting of many non-immune cells, we simulated the bulk gene expression profiles of tissues by in silico spiking the expression signals of immune cells into the microarray data of breast tissues. The microarray sample of three breast tissues, GSM739223-GSM739225, were downloaded from NCBI GEO and normalized by using the justRMA function of R package affy. In the simulated samples, three different proportions of the gene expression signals from breast tissues, namely, 30, 50 and 70% were tested. The expression profiles of the 9 samples for the immune cells to be in silico spiked into the simulated bulk breast tissue were randomly sampled and weighted from the 148 microarrays as shown in Table 1. In each test run, the deconvolution was performed by taking the remaining 139 microarrays to build the reference gene expression signature matrix for testing. To reveal the agreement between the predictions made by our method and the ground truths, Bland-Altman (BA) plots were generated in order to compare the prediction-truth agreements between CIBERSORT and our method. The cumulative percentages of observations for the difference between predictions and real values were tabulated in order to facilitate the comparison of performances between our method and CIBERSORT. Besides, mean absolute percentage errors (MAPEs) were calculated to measure the percentage of errors of the predicted cell fractions relative to the real cell-specific weights in the simulated bulk tissues. Box plots were produced to show the distributions of MAPEs and the results were compared with the MAPEs of the predictions made by CIBERSORT.

### Analysis of human PBMCs with flow cytometry results

On the other hand, the performance of our method was assessed by using real mixture gene expression profiles derived from PBMCs. Three sets of microarray samples of human PBMCs, including GSE65133 [11], GSE106898 [22], and GSE107990 [22], were downloaded from NCBI GEO, and the three datasets contain 20, 12, and 164 microarray samples, respectively. Since each of these PBMC samples has been determined for its composition of immune cells by using flow cytometry, the agreement of the cell abundance predicted by our method with the experimentally determined composition could be measured. The platform of those microarray samples is the Illumina HumanHT-12 V4.0 expression beadchip, and the raw data were quantile-normalized by using the R package preprocessCore. Because our RefGES was created by using the expression profiles generated by Affymetrix U133 Plus 2.0 platform, there was an issue about inconsistent distribution of expression levels when data generated by non-Affymetrix platforms were used. Thus, we followed a simple cross-platform quantile normalization approach as used in [23]. We took the 148 microarrays used in this study as the reference set, and then the expression profiles generated by the Illumina platform were transformed to make their empirical cumulative distribution function similar to the reference set. MAPEs were calculated, BA plots were generated, and cumulative percentages of observations for the difference between predictions and real values were tabulated to compare the performances of our method and CIBERSORT.

## Results

### Building the reference gene expression signature matrix

**Fig. 2:**
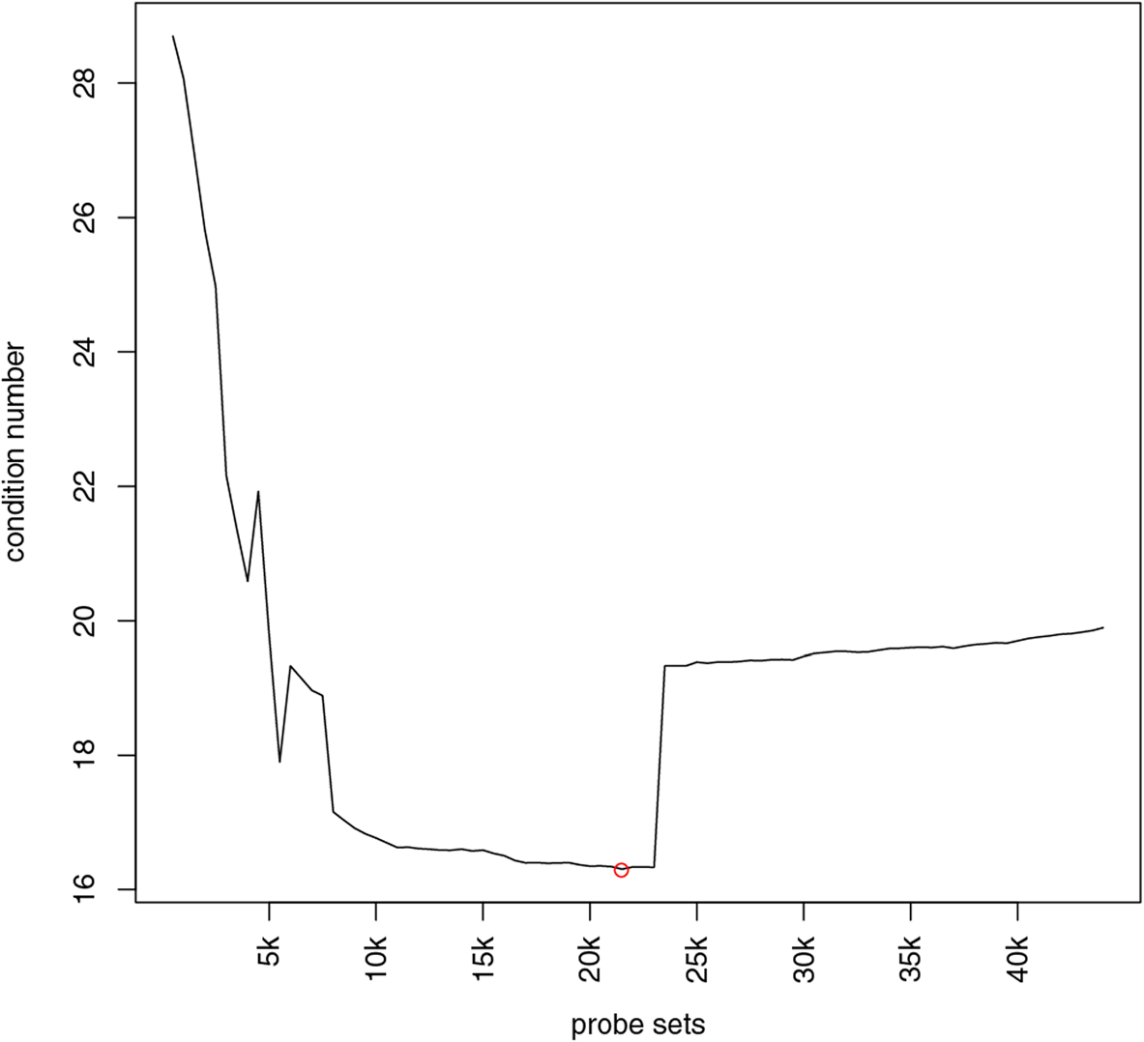
The condition numbers for the top lists of the DEGs ranked by *P*-value. Red circle is to indicate the location of the minimum condition number, where the number of probe sets is 21,500

**Fig. 3:**
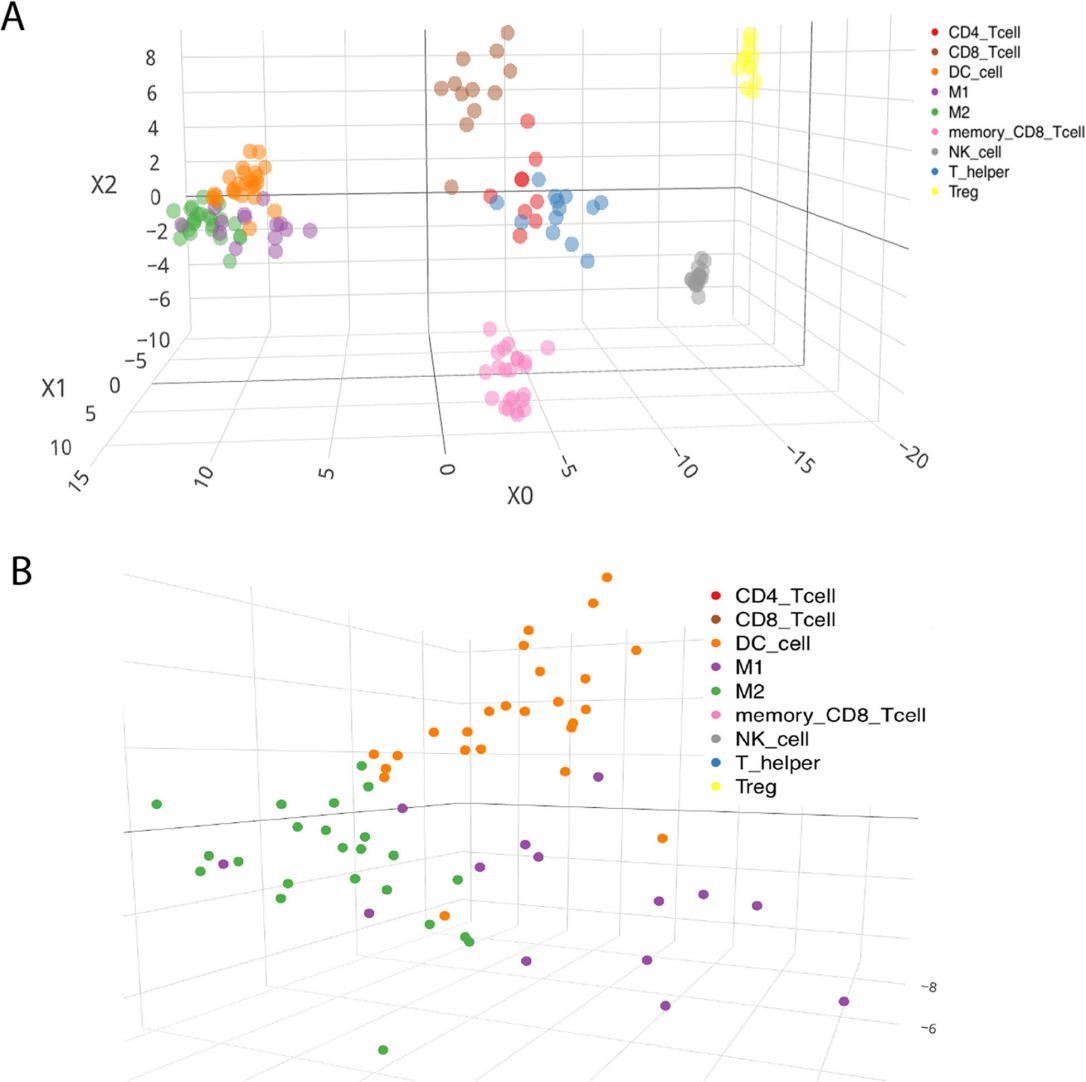
The LDA plot of the 148 immune cell samples using the expression data of SGs. **a** The 3D LDA plot for all the samples. **b** The zoom-in view of the LDA plot for the samples of M1, M2, and DC cells

Of the 54,675 probe sets in the Affymetrix GeneChip Human Genome U133 Plus 2.0 Array, 44,224 probe sets were found to be differentially expressed across 9 types of immune cells in the ANOVA of the 148 microarray samples for building the reference gene signature. By using the approach of minimizing the condition number as mentioned, it was determined that the optimal *G* value was 21,500 (Fig. 2), corresponding to 12,366 genes, including 11,114 protein coding genes and 1252 non-protein coding genes. The gene expression matrix of 12,366 genes × 148 arrays was further collapsed to a matrix of 12,366 genes × 9 immune cell types by taking the median value of the expression levels of each gene within each type of immune cells. This matrix was used as the reference gene expression signature matrix (RefGES) in this study. The RefGES matrix is provided in Additional file 2: Table S2. Those 12,366 genes as a whole will be described in the following text as the signature genes (SGs). To reveal the global structure of the data [24], these SGs were used to perform a linear discriminant analysis (LDA) and the first three axes that can maximize the separation between multiple classes were taken to generate a three-dimensional (3D) plot for visualization (Fig. 3a). In general, different types of cells could be distinctly separated from the others by rotating the 3D LDA plot, except for that 3 samples of M1 cells and 2 samples of dendritic cells clearly located outside of their respective clusters (Fig. 3b). Besides, by using WGCNA, the 148 cells could be clustered into nine groups, each group containing cells exclusively belonging to a single type of immune cells.

### Concordance between the predicted cell composition and the true proportions

#### Pure-cell samples

Our method was then assessed by using the gene expression profiles with known proportions of immune cells. First, each of the 148 microarray samples was tested by using a leave-one-out strategy as mentioned in the Materials and Methods. Since each microarray sample was derived from only a single type of immune cell, these microarray samples were labeled as pure-cell samples, and the true cell composition for each of the pure-cell samples is 1.00 for its distinctive type of immune cell.

**Fig. 4:**
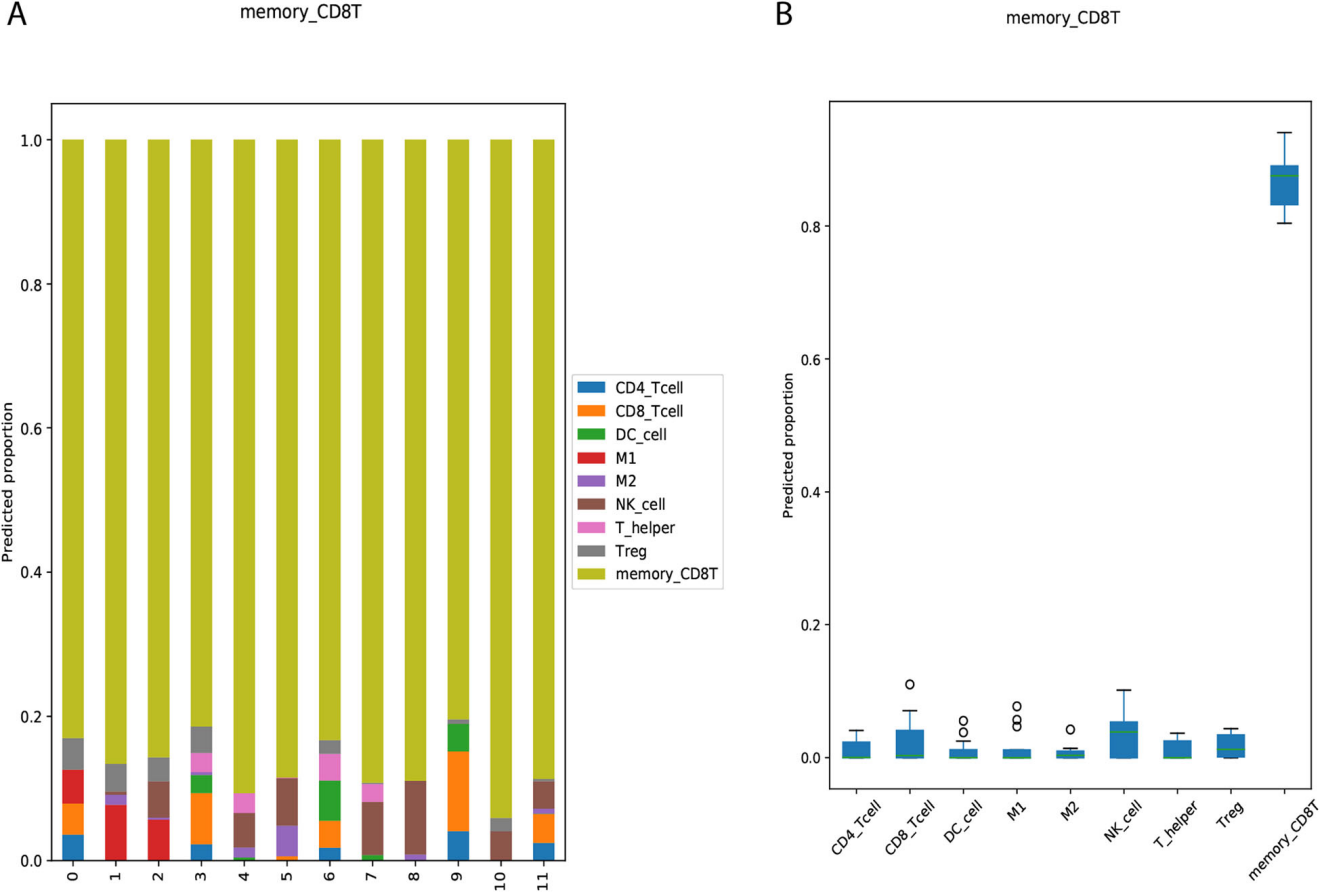
The (**a**) bar charts and (**b**) box plots of the predicted cell fractions for the pure-cell samples consisting of only memory CD8 T cells (denoted as memory_CD8T in the figures)

In this benchmark, the prediction made by our method could recover the major cell composition in the pure-cell samples. For example, for all of the 12 pure-cell samples consisting of only memory CD8 T cells (Fig. 4), our method consistently predicted that memory CD8 T cells were the dominant type of immune cells (> 0.8), and the predicted fractions for other cell types were mostly lower than 0.10. In addition, the prediction made by our method also agreed well with the ground truth for the other 8 types of cells, as illustrated in Additional file 1: Figure S1.

The results of this benchmark are summarized in Table 2. Our method achieved a mean value of at least 0.81 for each type of pure-cell microarray samples, whereas the mean cell fractions predicted by CIBERSORT were much lower for each type of pure-cell sample. In addition, the cell fractions predicted by CIBERSORT also showed a higher variation when deconvoluting the pure-cell samples of DC, M2, NK, and CD4 T cells, than those predicted by our method. Among the 9 types of pure-cell samples, the performance of CIBERSORT was the worst for M1 cells, T helper cells, Treg cells, and memory CD8 T cells, where the mean predicted fractions for them were 0.0, ~ 0.05, ~ 0.09, and ~ 0.0005, respectively. To sum up, for this benchmark CIBERSORT failed to recover the dominant cell type from the gene expression profiles of pure-cell samples for M1 cells, T helper cells, Treg cells and memory CD8 T cells, respectively.

**Table 2:** The means and standard deviations (SD) of the predicted cell fractions by our method and by CIBERSORT, respectively, using the 148 pure-cell samples

	Our method		CIBERSORT	
	Mean	SD	Mean	SD
DC	0.85	0.09	0.24	0.33
M1	0.81	0.07	0	0
M2	0.84	0.14	0.38	0.38
NK	0.81	0.11	0.26	0.37
CD4 T	0.82	0.08	0.71	0.19
Th	0.87	0.09	0.05	0.04
Treg	0.87	0.05	0.09	0.06
CD8 T	0.85	0.11	0.27	0.10
Memory CD8 T	0.87	0.04	0.0005	0.001

### In silico cell mixtures and simulated bulk tissues

**Fig. 5:**
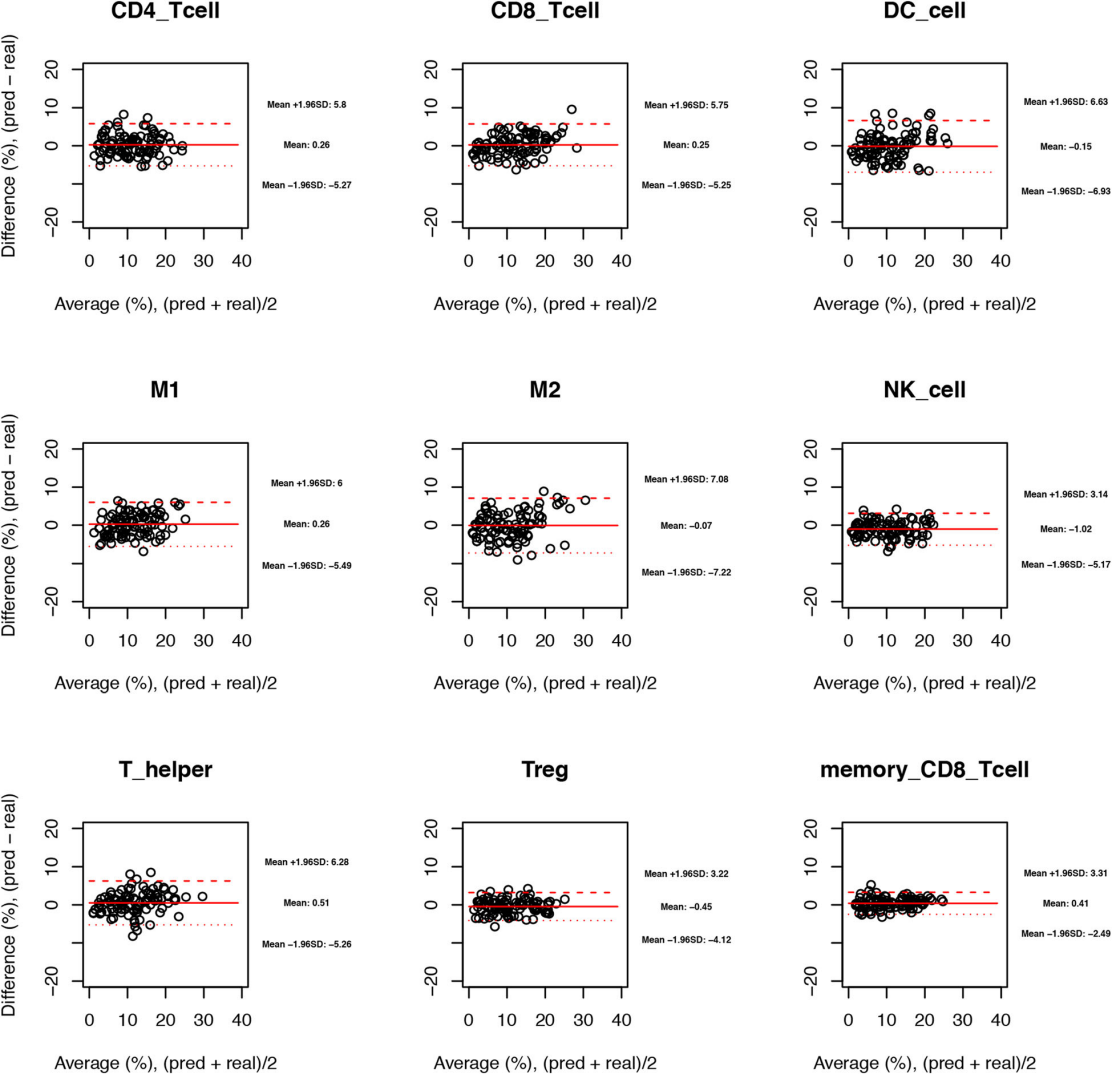
The BA plots showing the agreement of predicted cell fractions with the real cell compositions of in silico pure-cell mixtures. CD4_Tcell and CD8_Tcell correspond to naïve CD4 T cells and naïve CD8 T cells, respectively

To evaluate if our method might recover the cell composition by taking the expression profiles of cell mixtures, we prepared in silico mixtures of immune cells and simulated bulk tissues. First, one hundred cell mixtures were generated, and each mixture with a randomly assigned fraction. It turns out that for each of the 9 immune cell types the predicted cell fractions had high agreements with the true cell proportions (Fig. 5), since all of the 95% limits of agreement (LoA) in the BA plots were small (mean of difference = 0, all of the LoAs are within the range of − 8 and 8%).

**Fig. 6:**
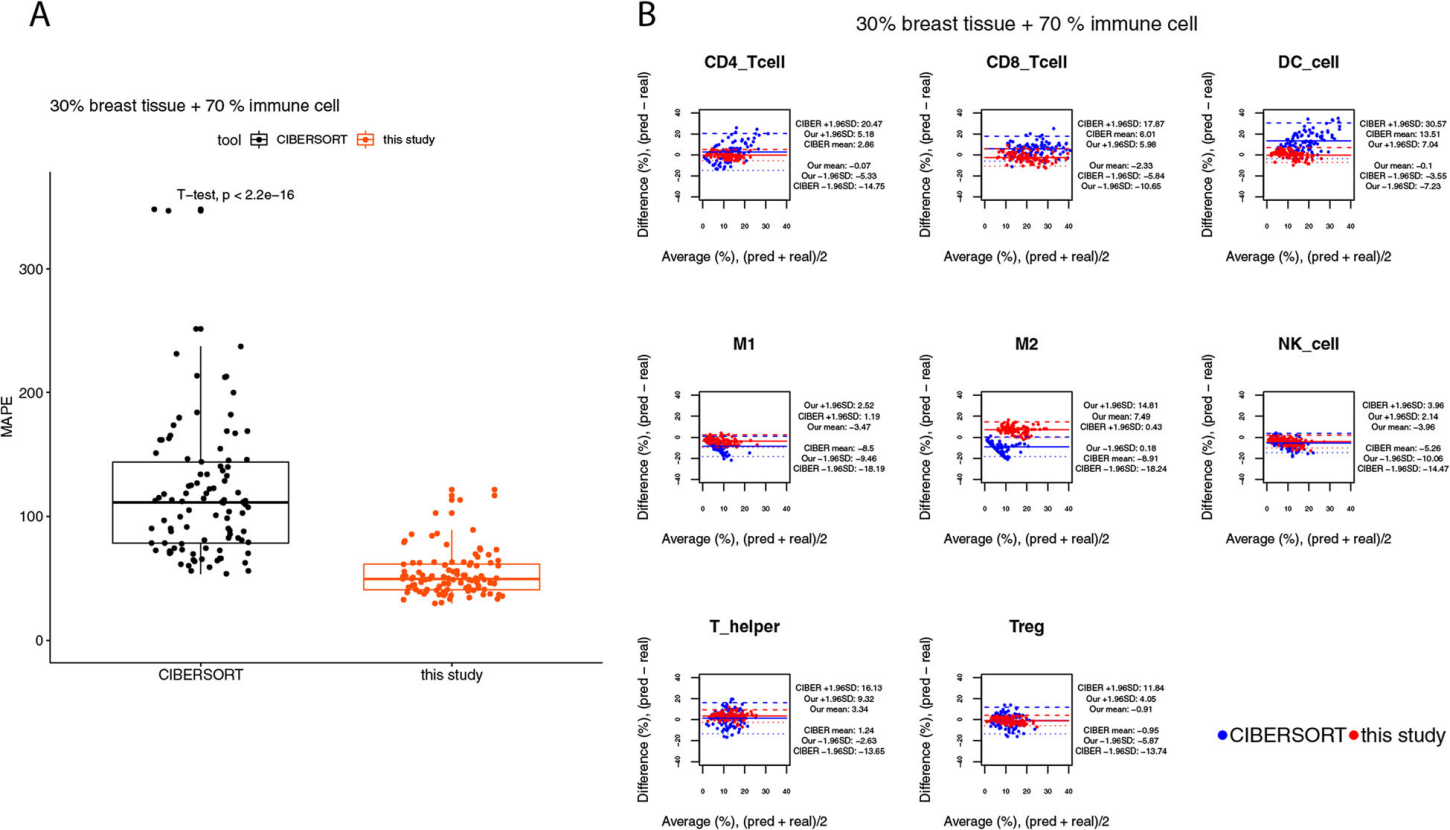
Agreement analysis for the predicted cell fractions with the real cell composition, in the simulated bulk tissues with 30% expression levels from breast tissues. **a** The box plot for the mean absolute percentage errors (MAPEs) at the sample level. **b** The BA plots for the agreement for each cell type in each of the samples

Next, simulated bulk tissues were created, where 30, 50%, or 70% of the convoluted expression levels were from breast tissues, and the remaining part was composed of the 9 types of immune cells with randomly assigned weights. For a performance comparison, the same set of expression profiles of simulated bulk tissues were used for cell composition prediction by CIBERSORT and by our method. For the benchmark of using the simulated bulk tissues containing 30% expression levels from breast tissues and 70% from immune cells, the result suggests that, across all of the 9 types of immune cells, our method might have higher accuracy than CIBERSORT. The median value of the mean absolute percentage errors (MAPEs) of the predictions made by our method and the true values were 59% at the sample level. Our method might recover the cell fractions in each simulated bulk tissue more accurately and more consistently than CIBERSORT, as revealed by the lower median value and the narrower interquartile range (IQR) of the MAPEs estimated for the predictions made by our method (t-test, *p*-value < 0.001) (Fig. 6a). The cumulative percentages for the prediction-truth difference also reveal a trend that the predictions made by our method were less deviated from the real values, as compared to the predictions made by CIBERSORT (see Additional file 2: Table S3). The BA plots suggest that there might be a higher level of agreement of the predictions made by our method with the ground truth, since the widths of the limits of agreement (LoA) of our method were smaller than those of CIBERSORT (Fig. 6b). In addition, the BA plots for the predictions made by CIBERSORT show evidence of increasing variability of differences with increasing the average of the predicted and real cell compositions (Fig. 6b).

**Fig. 7:**
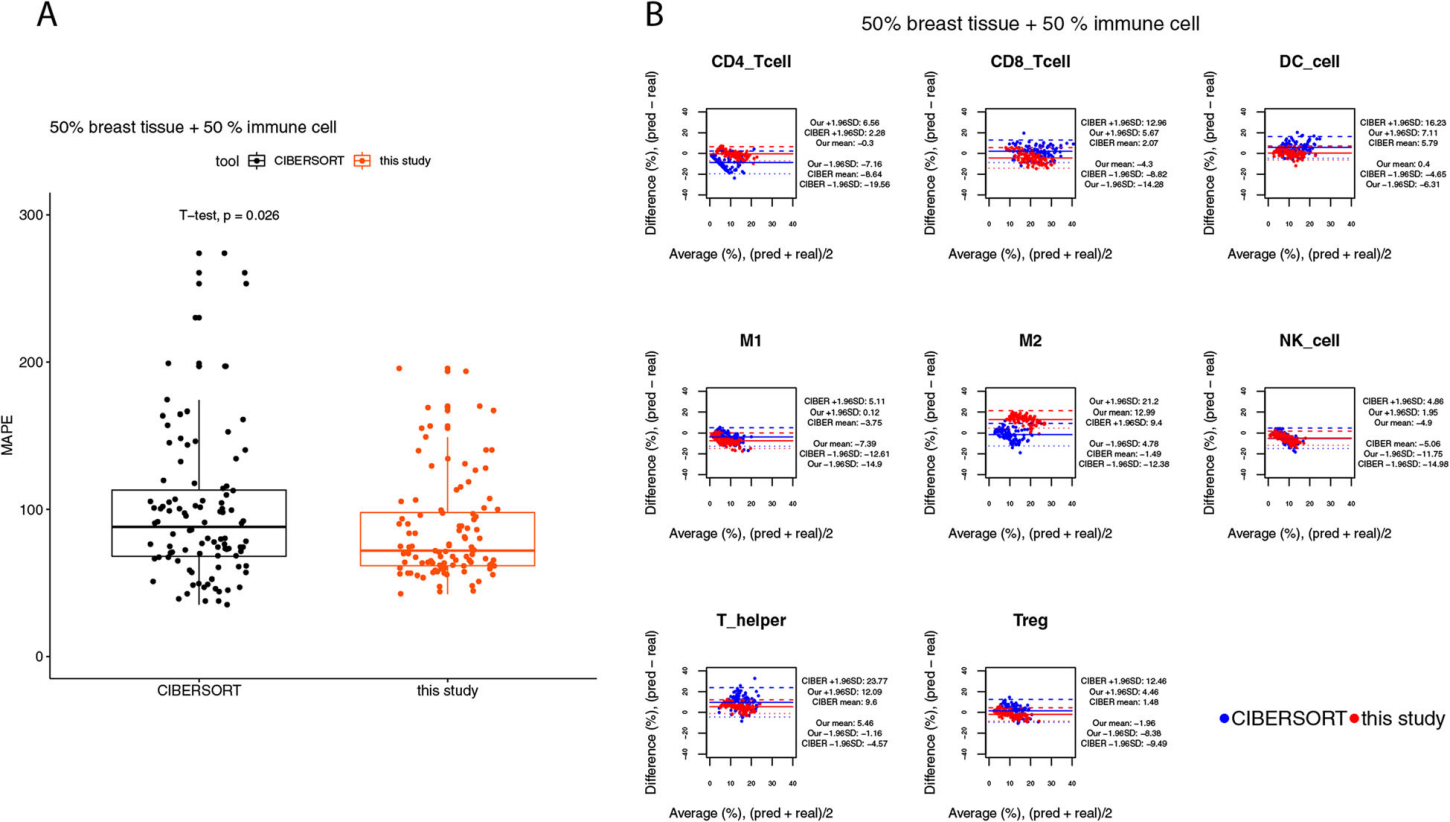
Agreement analysis for the predicted cell fractions with the real cell composition, in the simulated bulk tissues with 50% expression levels from breast tissues. **a** The box plot for the mean absolute percentage errors (MAPEs) at the sample level. **b** The BA plots for the agreement for each cell type in each of the samples

**Fig. 8:**
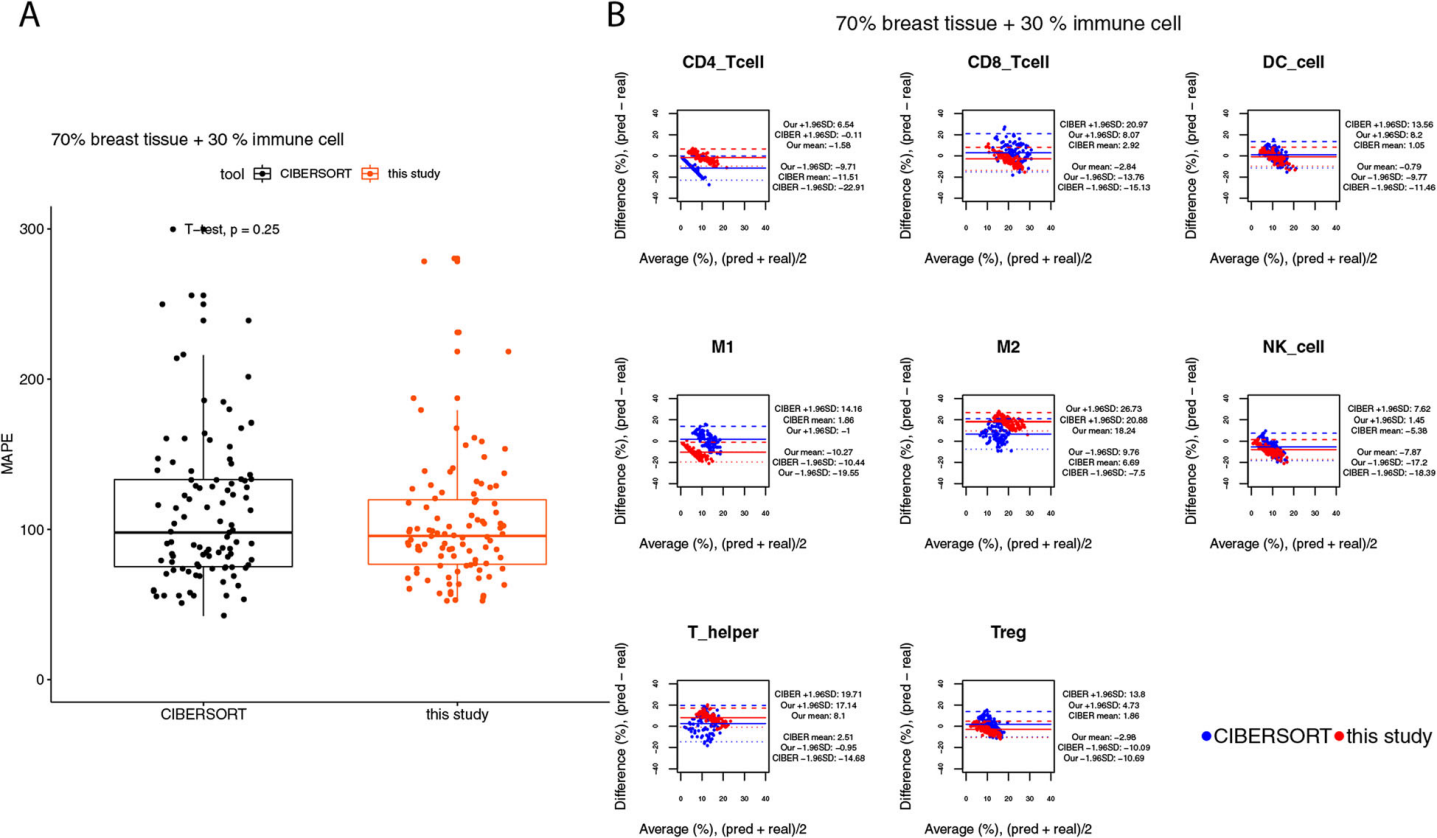
Agreement analysis for the predicted cell fractions with the real cell composition, in the simulated bulk tissues with 70% expression levels from breast tissues. **a** The box plot for the mean absolute percentage errors (MAPEs) at the sample level. **b** The BA plots for the agreement for each cell type in each of the samples

By using the simulated bulk tissues containing less than 70% expression levels from immune cells, we noticed that the performance of our method degraded obviously (see the changes from Figs. 6 and 7 to Fig. 8). When only 30% of the expression levels of the simulated bulk tissues were derived from immune cells, the median values of MAPEs were increased to 96% at the sample level (Fig. 8a). The performances of CIBERSORT and our method were not statistically different at the sample level (Fig. 8a, t-test, *p*-value = 0.25). The BA plot also shows evidence of increasing variability of differences, with increasing the average of the predicted and true cell compositions, for both of the predictions made by CIBERSORT and by our method (Fig. 8b). Nonetheless, the BA plots for this benchmark reveal that our method might be better in predicting the fractions for naïve CD4 T cells, CD8 T cells, dendritic cells, NK cells, and Treg cells, since for these cell types the widths of the limits of agreements (LoAs) of our method were smaller than those of CIBERSORT (Fig. 8b).

### The human PBMCs with flow cytometry results

Unlike the previous evaluations of our method where only pure-cell samples and in silico cell mixtures and simulated bulk tissues were used, the next set of benchmarks were based on real human samples consisting of multiple cell types — the human PBMCs with flow cytometry results. The purpose of these benchmarks was to evaluate if our method might still perform well when samples derived from real human tissues were analyzed. However, one consideration about this assessment is that our method was not designed to predict the fractions of all of the cell types that have been determined for the human PBMC samples. For example, only 4 out of the 9 cell types in the PBMC samples of GSE65133 could be treated by our method. The 9 cell types of the PBMC samples of GSE65133 are naïve B cells, memory B cells, CD8 T cells, naïve CD4 T cells, resting memory CD4 T cells, activated memory CD4 T cells, gamma delta T cells, NK cells, monocytes. Only naïve CD4 T cells, CD8 T cells and NK cells matched exactly with the types of immune cells that our method could predict their fractions in a mixture.

Therefore, in order to slightly expand the cell types that our method could treat for this benchmark, the fractions predicted by our method for macrophage M1 and M2 cells, as well as dendritic cells, were summed up as a means to estimate the fraction of the monocytes in the samples. This approach was used because monocytes can enter tissues and then be induced to differentiate into M1/M2 cells and dendritic cells, and those types of cells might still reserve certain gene expression signature of monocytes [25]. Besides, the fractions predicted for naïve CD4 T cells, T helper cells, and Treg cells would be summed up to estimate the fraction for CD4 T cells in the PBMC samples. Additional file 2: Table S6 is provided to reveal the aforementioned cell type mapping from the PBMCs to cell types of LM22 and the RefGES in this study.

**Fig. 9:**
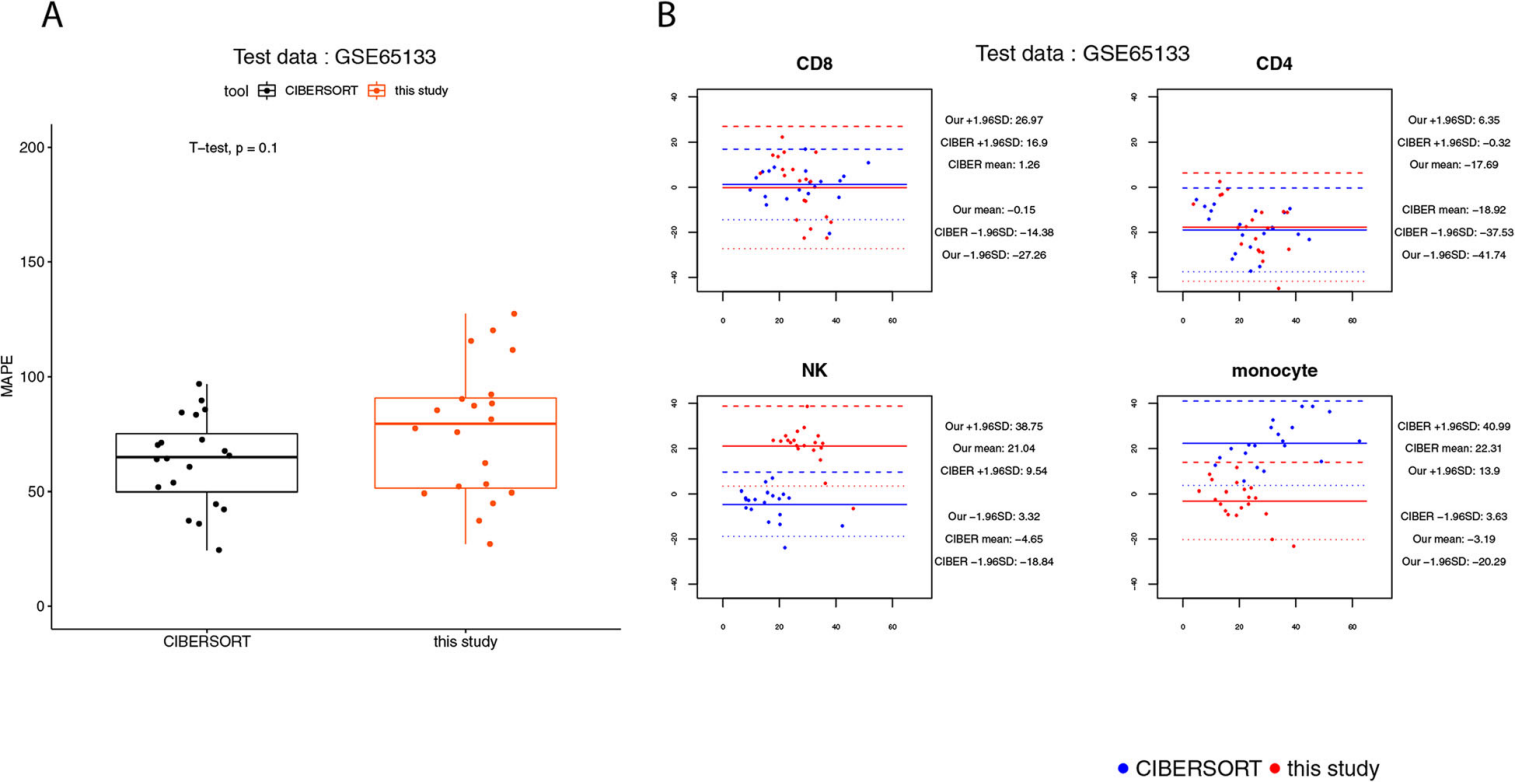
Agreement analysis for the predicted cell fractions with the real cell composition, in the 20 human PBMC samples of NCBI GEO GSE65133. **a** The box plot for the mean absolute percentage errors (MAPEs) at the sample level. **b** The BA plots for the agreement for each cell type in each of the samples

Thus, for the benchmark using GSE65133, the cell fractions predicted by our method for naïve CD8 T cells, CD4 T cells, natural killer cells, and monocytes were normalized to a sum of 1. The results of this benchmark suggest that there was no significant difference in the MAPEs of the predictions between CIBERSORT and our method (Fig. 9a). The predictions made by our method for monocytes might have a better agreement with the real values (Fig. 9b and Additional file 2: Table S7), although for the other cell types the widths of LoAs of our method were larger than those of CIBERSORT (Fig. 9b).

**Fig. 10:**
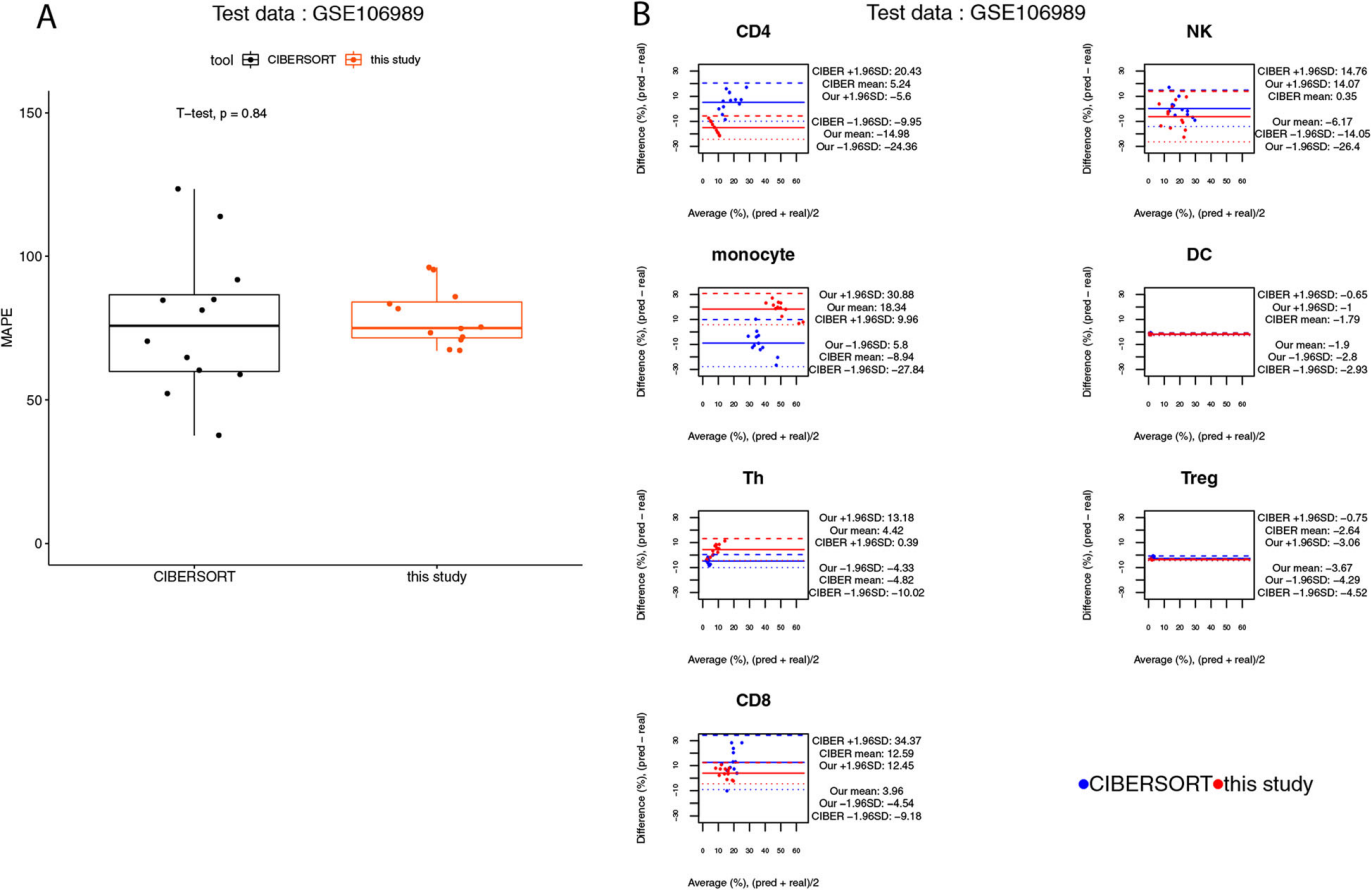
Agreement analysis for the predicted cell fractions with the real cell composition, in the 12 human PBMC samples of NCBI GEO GSE106898. **a** The box plot for the mean absolute percentage errors (MAPEs) at the sample level. **b** The BA plots for the agreement for each cell type in each of the samples

On the other hand, for the benchmark using the 12 PBMC samples of NCBI GEO GSE106898, the flow cytometry confirmed data contains the cell composition of 29 types of immune cells. In order to assess our method by using this dataset, a mapping table was created such that the cell composition of certain subtypes of immune cells were combined in order to assess its agreement with the predicted fraction of either one of the 7 cell types that could be treated by our method (see Additional file 2: Table S8 for the mapping table). The results of this benchmark suggest that there was no significant difference in the MAPEs of the predictions between CIBERSORT and our method (Fig. 10a). In this benchmark, the predictions made by CIBERSORT might have a higher level of agreement with real values than the ones made by our method, since the widths of LoAs of our method were larger for NK cells and T helper cells (Fig. 10b). The cumulative percentages of observations for the prediction-truth difference also reveal that the predictions made by our method were more deviated from the real values for these cell types (see Additional file 2: Table S9).

**Fig. 11:**
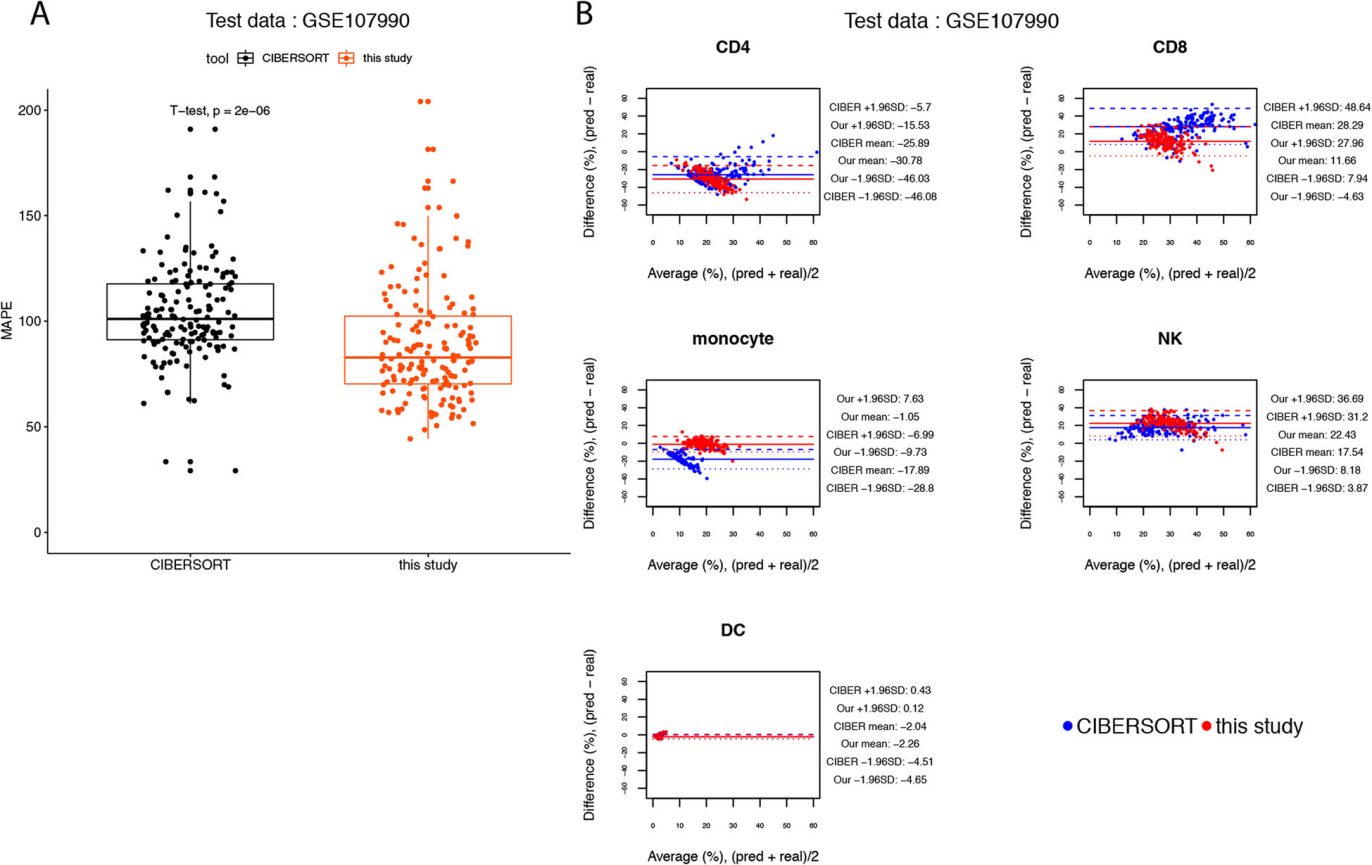
Agreement analysis for the predicted cell fractions with the real cell composition, in the 164 human PBMC samples of NCBI GEO GSE107990. **a** The box plots for the mean absolute percentage errors (MAPEs) at the sample level. **b** The BA plots for the agreement for each cell type in each of the samples

To further evaluate our method, another PBMC dataset GSE107990, which contained 164 samples, were downloaded from NCBI GEO database. The mapping between the 11 types of immune cells in this dataset and the 9 cell types in this study is given in Additional file 2: Table S10. In this benchmark, the MAPEs of the predictions made by our method was significantly lower than those predicted by CIBERSORT at the sample level (t-test, *p*-value < 0.001) (Fig. 11a). The BA plots show evidence of increasing variability of differences, with increasing the average of the predicted and true cell compositions, for both of the predictions made by CIBERSORT and by our method (Fig. 11b). Nevertheless, the predictions made by our method might have a better agreement with the ground truth for CD4 T cells, CD8 T cells, monocytes, and dendritic cells, since for the 4 types of cells the widths of LoAs of our method were smaller than those of CIBERSORT (Fig. 11b), which is also supported by the cumulative percentages of observations for the prediction-truth difference (see Additional file 2: Table S11).

## Discussion

We developed a cell composition deconvolution method that could use the gene expression profiles of complex samples to predict the cell fractions. Our method was designed to focus on the types of immune cells that have been suggested to influence the prognosis of cancers. There are several unique features that make our method very different from the other tools designed for the same purpose.

First, for each immune cell type to be analyzed in this study, at least 9 replicates of microarrays were recruited to build our reference gene expression signature (RefGES) (Table 1). For example, we recruited 16 replicates for Treg cells, 14 replicates for M1 cells, and 25 replicates for M2 cells. Since the general approach to creating a reference expression profile matrix is to take the median value of the expression levels of a gene across all the samples of a single cell type, between-sample variations for a probe set due to technical limitations might have a certain impact on the robustness of the RefGES, which might in turn influence the performance of the cell deconvolution methods. Therefore in this study, more replicates for each cell type were included in an attempt to create a more robust RefGES matrix for the use of regression-based deconvolution.

Second, since the microarray platform of the samples for building our RefGES is Affymetrix U133 plus 2, this means that not only protein-coding genes but also non-coding RNA genes might be included in the reference gene expression signature (RefGES) that would be used to perform cell composition deconvolution. For example, among the probe sets of Affymetrix U133 plus 2, there are 6492 probe sets corresponding to 5563 lncRNAs [26]. We know that the number of human non-coding RNA genes is still growing, and to date more non-coding RNA genes than protein-coding genes have been annotated in the human genome (for the statistics see [27]). In a modern RNA-seq experiment, it is quite common that ~ 10 k to ~ 20 k non-coding RNAs would be sequenced. Thus, including non-coding RNA genes in a RefGES for cell composition deconvolution appears to be a natural choice if a deconvolution method is to take RNA-seq data sets as the input.

Third, ANOVA was used in this study to choose differentially expressed genes across different types of immune cells. By contrast, other available cell deconvolution methods have selected their immune signature genes by recruiting well-known cell specific markers [28] or by applying a pairwise t-tests approach to gather differentially expressed genes [11]. Our gene selection approach leads to the inclusion of ~ 13 k genes in the RefGES. This number is ~ 23 times greater than the genes recruited in the reference profile, LM22, that was created and used by CIBERSORT [11]. At first glance, it seems suspicious that including so many genes in this method is likely to incorporate genes with outlier expression levels and thus introduce more noise into the RefGES. Nonetheless, the benchmarks of our method reveal that our method along with the reference profiles might have a performance at least comparable to the state-of-the-art deconvolution method, CIBERSORT. In addition, our method might have the merit that the agreement of the predicted cell composition with the ground truth did not degrade in a monotonously increasing manner as the magnitude of the variable of interest increases, which is supported by the BA plots for the benchmark of using simulated bulk tissues with 70 and 30% of expression levels from immune cells and breast tissues, respectively, and by the BA plots for the benchmark of using the PBMC samples of NCBI GEO GSE107990.

There are several limitations on the current results of our method. Due to the limited sources of testing data sets derived from real complex samples, our method has been evaluated mainly by using expression profile samples composed of simple pure-cell types, in silico cell mixtures and simulated bulk tissues. The agreement analyses in the benchmarks using simulated bulk tissues suggest that the performance of our method is likely to degrade by increasing the level of contribution of non-immune cells to the bulk gene expression profiles. However, it is difficult to further investigate this issue by using real samples, since to the best of our knowledge no public-domain bulk gene expression profiles with experimentally confirmed cell composition are derived from real human complex tissues such as tumor masses, which might contain abundant non-immune cells as well as immune cells.

On the other hand, the only real complex samples used for the benchmarking in this study are the three sets of microarrays of human PBMC samples with flow-cytometry confirmed cell composition results. However, our method could predict the fractions of only a subset of the cell types discovered in the human PBMC datasets, since our RefGES matrix does not include the reference expression profiles for all of the cell types in the PBMC samples. This means that these PBMC-based benchmarks could just partially assess the performance of our method in terms of the scope of immune cell types, since the accuracy of our method in predicting the fractions of M1 and M2 cells could not be evaluated based on real complex samples. The PBMC cell types missed by our method may still influence the performance of our method, since the gene expression signals of the inevitably missed cell types might be falsely deconvoluted into the 9 types of immune cells that could be analyzed by our method. Nonetheless, this limitation is also one of the bottlenecks that each cell composition deconvolution method may encounter, since benchmarking with the gene expression profiles derived from real complex tissues is most critical to demonstrate the accuracy of such methods, and it is difficult to acquire a decent dataset of gene expression profiles with confirmed fractions of as many cell types as possible. After all, our method has been designed to focus on only the 9 types of immune cells that might be associated with cancer immunity and patients’ prognosis. This design has also limited the benchmarking, making us unable to conduct a completely fair comparison of the performance of our method with the available methods.

One topic that is beyond the current scope of this study is the cell composition deconvolution of the RNA-seq data derived from bulk tissues. Our method has been developed by recruiting microarray-derived transcriptomic profiling data of 9 types of immune cells to build the RefGES. Consequently, to use our method to analyze bulk-tissue RNA-seq data, non-trivial data normalization approaches must be tried in order to transform read counts to fit the distribution characteristics of the reference microarray data [3, 29]. By contrast, an alternative approach to performing cell composition deconvolution of RNA-seq data of bulk tissues is to build the reference gene expression signatures by using the RNA-seq data derived from the cell types of interest for different research aims.

Recently several studies have utilized cell-type specific gene expression profiles derived from single-cell RNA-sequencing (scRNA-seq) technologies for cell composition deconvolution [30, 31]. MuSiC is one of such methods ad hoc designed to take advantage of scRNA-seq data as the reference for cell-type deconvolution [30]. The underlying rationale of the development of such methods is that scRNA-seq is likely to provide cell type-specific gene expression profiles, facilitating the build of a robust gene-expression reference for different cell types. Interestingly, MuSiC uses a tree-guided approach that allows an iteratively zoom-in deconvolution, which may remedy the multicollinearity problem when certain cell types of interest have very similar gene expression profiles. Therefore, MuSiC outperforms other cell composition deconvolution methods in analyzing the gene expression data derived from bulk renal tissues [30]. However, there are concerns with respect to applying these methods to analyze bulk RNA-seq data derived from complex tissues other than kidney. For example, for deconvoluting the expression profiles derived from one type of tissue, MuSiC requires that the reference scRNA-seq data is derived from the same tissue type, or from a population with a similar abundance distribution of the cells involved in this tissue type [30]. This requirement might not be very practical since in complex tissues such as tumors the types of infiltrating cells as well as their relative abundance might be varied from one sample to the others. In addition, apart from the major cell types in renal tissues, only three types of immune cells have been treated in the benchmarks of MuSiC. Hence, it is unclear whether MuSiC might be applicable to deconvolute more than 3 types of immune cells from the RNA-seq data of complex tissues. Nevertheless, deconvoluting cell composition from bulk-tissue RNA-seq data is definitely one of the central topics worthy of further exploration, since RNA-seq has been widely adopted to explore the transcriptomic features in different disease states, including cancers. To date, thousands of RNA-seq datasets derived from bulk samples of tumors in more than 30 types of cancers have been provided by public resources such as The Cancer Genome Atlas (TCGA) project [32], and improving cell composition deconvolution methodologies in order to analyze bulk-tissue RNA-seq data is an important direction for future development.

## Conclusions

The deconvolution method created in this study is able to take bulk expression profiles to predict the fractions of 9 types of immune cells. The benchmark of our methods showed that the performance of our method might be superior to that of other methods being developed for the same purpose. The source code of our method could be downloaded from https://github.com/holiday01/deconvolution-to-estimate-immune-cell-subsets.

## Supplementary information

**Additional file 1: Figure S1.** The bar charts and box plots of the predicted cell fractions for the pure-cell samples. (A) and (B) for samples of naïve CD4 T cells, (C) and (D) for samples of naïve CD8 T cells, (E) and (F) for dendritic cells, (G) and (H) for macrophage M1 cells, (I) and (J) for macrophage M2 cells, (K) and (L) for natural killer cells, (M) and (N) for T helper cells, (O) and (P) for regulator T cells.

**Additional file 2: Table S1.** The microarray samples downloaded from NCBI GEO as the raw data to be used as the reference gene expression profiles, each being annotated with its source NCBI GEO sample accession numbers (GSM). **Table S2.** The reference gene expression signature (RefGES) matrix created in this study. **Table S3.** The cumulative percentages of observations for the difference between predictions and real values in the benchmark using the simulated bulk tissues with 30% expression levels from breast tissues and 70% from immune cells. **Table S4.** The cumulative percentages of observations for the difference between predictions and real values in the benchmark using the simulated bulk tissues with 50% expression levels from breast tissues and 50% from immune cells. **Table S5.** The cumulative percentages of observations for the difference between predictions and real values in the benchmark using the simulated bulk tissues with 70% expression levels from breast tissues and 30% from immune cells. **Table S6.** The mapping of the cell types of NCBI GEO GSE65133 to those of LM22 (CIBERSORT) and the RefGES used in this study. **Table S7.** The cumulative percentages of observations for the difference between predictions and real values in the benchmark using the 20 human PBMC samples of NCBI GEO GSE65133. **Table S8.** The mapping of the cell types of NCBI GEO GSE106898 to those of LM22 (CIBERSORT) and the RefGES used in this study. **Table S9.** The cumulative percentages of observations for the difference between predictions and real values in the benchmark using the 12 human PBMC samples of NCBI GEO GSE106898. **Table S10.** The mapping of the cell types of NCBI GEO GSE107990 to those of LM22 (CIBERSORT) and the RefGES used in this study. **Table S11.** The cumulative percentages of observations for the difference between predictions and real values in the benchmark using the 164 human PBMC samples of NCBI GEO GSE107990.

## Data Availability

All of the source datasets downloaded from NCBI GEO for building the reference gene expression signature (RefGES) matrix are listed along with their GEO sample accessions numbers (GSM) in Additional file 2: Table S1. The RefGES matrix generated in this study is shown in Additional file 2: Table S2.

## Abbreviations

3D: Three-dimensional
ANOVA: Analysis of variance
BA: Bland-Altman
CI: Confidence interval
DC: Dendritic cell
DEG: Differentially expressed gene
GEO: Gene Expression Omnibus
IHC: Immunohistochemistry
IQR: Interquartile range
LDA: Linear discriminant analysis
lncRNA: Long non-coding RNA
LoA: Limits of agreement
MAE: Mean average error
MAPE: Mean absolute percentage error
NCBI: National Center for Biotechnology Information
NK: Natural killer
PBMC: Peripheral blood mononuclear cell
RefGES: Reference gene expression signature matrix
RMA: Robust Multi-array Average
scRNA-seq: Single-cell RNA-sequencing
SD: Standard deviation
SG: Signature gene
SVR: Support vector regression
TCGA: The Cancer Genome Atlas
Th: T helper cell
TME: Tumor microenvironment
Treg: Regulatory T cell
WGCNA: Weighted gene co-expression network analysis
ε-SVR: ε-insensitive support vector regression
