# Supplementary figures and images for: Improved cell composition deconvolution method of bulk gene expression profiles to quantify subsets of immune cells
Source: BMC Med Genomics. 2019 Dec 20;12(Suppl 8):169. doi: 10.1186/s12920-019-0613-5 (PMC6923925; doi:10.1186/s12920-019-0613-5)

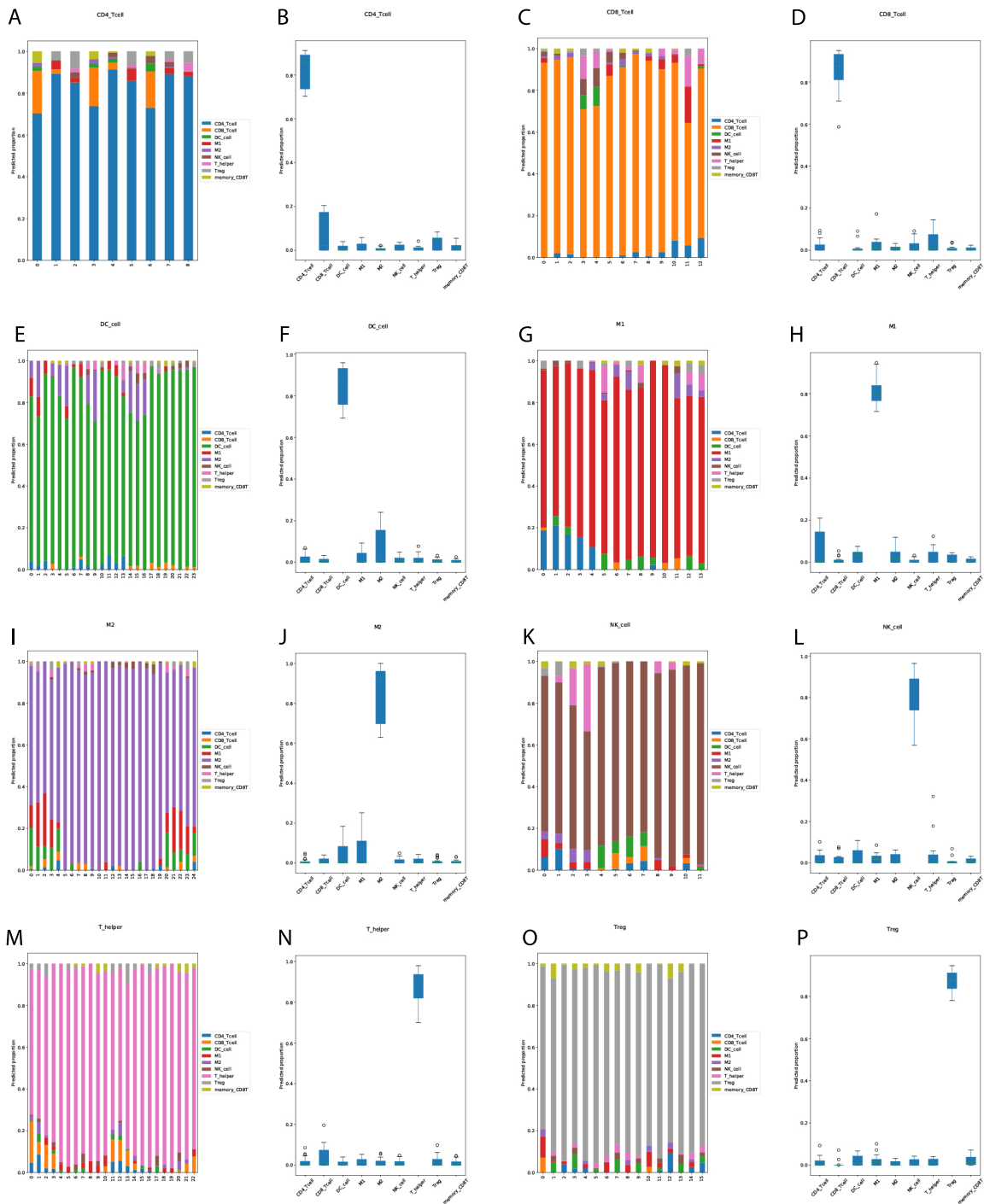

Supplement: Supplementary file 1 — Additional file 1: Figure S1. The bar charts and box plots of the predicted cell fractions for the pure-cell samples. (A) and (B) for samples of naïve CD4 T cells, (C) and (D) for samples of naïve CD8 T cells, (E) and (F) for dendritic cells, (G) and (H) for macrophage M1 cells, (I) and (J) for macrophage M2 cells, (K) and (L) for natural killer cells, (M) and (N) for T helper cells, (O) and (P) for regulator T cells. [file 12920_2019_613_MOESM1_ESM.pdf]
